# Chemometric-assisted spectrophotometric approach for stability assessment of safinamide and its synthetic precursor in antiparkinsonian formulation with sustainability profiling

**DOI:** 10.1038/s41598-025-28085-4

**Published:** 2025-11-29

**Authors:** Engy A. Ibrahim, Samah S. Saad, Maha A. Hegazy, Laila E. Abdel Fattah, Hoda M. Marzouk

**Affiliations:** 1 Pharmaceutical Analytical Chemistry Department, College of Pharmaceutical Sciences and Drug Manufacturing, Misr University for Science & Technology, 6th of October City, Giza Egypt; 2 Pharmaceutical Chemistry Department, Faculty of Pharmacy, Future University in Egypt, Cairo, 11835 Egypt; 3 Pharmaceutical Analytical Chemistry Department, Faculty of Pharmacy, Cairo University, Kasr Al-Aini Street, Cairo, 11562 Egypt

**Keywords:** Blueness assessment, Greenness assessment, 4-hydroxy benzaldehyde, Multivariate algorithms, PCR, PLS, Safinamide mesylate, Stability indicating methods, siPLS, Whiteness assessment, Chemistry, Drug discovery

## Abstract

Parkinson’s disease (PD) is a significant health issue that affects older individuals. Safinamide mesylate (SAF) is a recently developed adjunct therapy increasingly employed in the management of PD. Ensuring the stability of new drug formulations and establishing suitable stability-indicating methodologies is crucial for pharmaceutical analysis. The current study has developed three new, user-friendly, and robust mathematical methods using multivariate spectrophotometric analysis to quantify SAF, its synthetic precursor impurity (4-HBD), and stress-induced degradation products. The multivariate algorithms used include principal component regression (PCR), partial least squares (PLS), and synergy intervals partial least squares (siPLS). The ranges of the proposed methods were 3.00–23.00, 1.00–5.00, 2.50–6.50, and 5.00–13.00 µg/mL for SAF, 4-HBD, SAF hydrolytic degradation products, and SAF oxidative degradation products, respectively. When analyzed using the applied methods, the commercially available tablet preparation showed stability with no impurities or interference from tablet additives. In terms of accuracy and precision, the statistical analysis also revealed no significant differences in comparison to the reported methods. The developed multivariate models were validated using internal and external validation sets. The results demonstrated that the siPLS model outperformed PCR and PLS based on the root mean square error of prediction (RMSEP) and the correlation coefficient values (r). Furthermore, these methods can be used as a substitute for HPLC in quality control laboratories when multiple samples need to be analyzed within a short timeframe. Finally, various sustainability assessment tools were utilized to evaluate and measure the environmental background of the established methods.

**Supplementary Information:**

The online version contains supplementary material available at 10.1038/s41598-025-28085-4.

## Introduction

The hallmark of Parkinson’s disease (PD) is the degeneration of the substantia nigra’s dopaminergic neurons, which is accompanied by motor symptoms^1^. Levodopa is the gold standard for managing PD motor symptoms. Dopamine replacement therapy is the primary treatment for this disorder^2^. On the other hand, levodopa side effects for an extended time include wearing off and developing signs of dyskinesia^3^. Along with levodopa, additional anti-parkinsonian medications such as monoamine oxidase-B (MAO-B) inhibitors, dopamine agonists, and others are required as the disease progresses^4^.

Safinamide mesylate (SAF), also known as (S)-2-[[4-[(3-fluorophenyl)methoxy]phenyl] methyl] aminopropanamide methane sulfonate^5,6^. It is an innovative medication that exerts its effects on both dopaminergic and non-dopaminergic receptors^7^. It controls dopamine levels by inhibiting monoamine oxidase B (MAO-B) and decreasing glutamate release^8^. It has been approved by the United States Food and Drug Administration (FDA) and the European Medicines Agency (EMA) as an additional treatment for patients with PD in the advanced stages who are already using levodopa either alone or in combination with other medications^7,9^. The synthetic precursor of SAF is 4-hydroxybenzaldehyde (4-HBD)^10^.

The importance of impurity profiling has increased in the pharmaceutical sector over the past few decades. Even small amounts of impurities can affect both the safety and effectiveness of drugs^11^. Several regulatory authorities, including the FDA and the International Council on Harmonization (ICH), have established guidelines on purity requirements and the detection of impurities in active pharmaceutical ingredients^11^. Researchers who employ analytical methods encounter a considerable obstacle when conducting both qualitative and quantitative analyses of impurities in order to meet established criteria^12^.

Currently, multivariate calibration models, such as principal component regression (PCR) and partial least squares (PLS), are extensively employed for determining components in complex matrices, including biological fluids, environmental samples, and commercial dosage forms^13^. Additionally, chemometric models are employed to analyze various datasets, including UV-VIS spectra, infrared spectra, chromatograms, and voltammograms obtained from different analytical instruments. PCR and PLS are highly regarded chemometric models due to their ability to rapidly and accurately quantify complex drug combinations^14^. However, several research studies have hypothesized that the optimal subinterval selection in the siPLS approach may yield more accurate and reliable prediction results than the conventional PLS model, which is based on full-spectrum manipulation. The siPLS model chooses the best subregions to represent the model’s overall performance across a range of subintervals. It can assist in finding the optimal model by searching for every possible combination of subintervals^15^.

The principles of green chemistry primarily drive the promotion of sustainability in laboratory and industrial settings. Anatas has developed the twelve principles of Green Analytical Chemistry (GAC) to guide individuals interested in applying this strategy^16^. Experts in organic, analytical, and chemical engineering, as well as other disciplines, provide a framework for implementing guidelines to enhance the environmental friendliness of chemical materials and processes^17^. Many efforts to create more environmentally friendly chemical processes emphasize the use of less toxic and green solvents, eliminating solvent usage, and reducing the quantity of chemical reagents required. Furthermore, utilizing underivatized samples and raw materials obtained from renewable resources are two additional methods to conserve energy^18,19^.

The literature provides a comprehensive overview of various analytical methods for determining SAF, either individually or in combination with other drugs. These methods include UV-spectrophotometric^20^, spectrofluorimetric^19^, HPLC^21–26^, HPTLC^27^, in addition to potentiometric and voltammetric methods^28–31^. The review noted that only a few stability-indicating chromatographic reports quantify SAF and its synthetic precursor impurity (4-HBD) in the presence of stress-induced degradation products^10,32^. Due to the limitations of using numerous hazardous solvents, lengthy analysis times, and high solvent and energy consumption in the reported chromatographic methods. Multivariate-assisted spectrophotometric methods present a promising alternative to HPLC for the rapid and efficient quality control analysis of these compounds in pharmaceutical formulations^33^. Furthermore, the literature indicates that no chemometric methods have been reported for the simultaneous determination of the compounds under investigation .

In this study, novel and sustainable multivariate spectrophotometric approaches were applied to resolve complex spectral overlaps and achieve simultaneous quantification of SAF, its synthetic precursor impurity (4-HBD), and stress-induced degradation products. The proposed methods were designed for accurate analysis, utilizing UV spectral data of both the pure forms and the pharmaceutical formulation. Furthermore, a comparative analysis was performed among the proposed multivariate techniques. PCR and PLS serve as fundamental chemometric models in pharmaceutical data analysis, facilitating the selective extraction of meaningful information from complex spectral datasets^34^. The siPLS model further advances analytical performance by optimizing variable selection to enhance signal interpretation and model accuracy^35^. These models are well-suited for the routine analysis of SAF and can assist pharmaceutical industries in conducting stability studies for the proposed drug across various formulations. Additionally, a significant advantage is their development using cost-effective and readily available instrumentation and software.

## Experimental

### Apparatus and chemicals

The UV-1650 PC double-beam UV-visible spectrophotometer (Shimadzu, Kyoto, Japan) was utilized for the spectrophotometric measurements. A 1 cm quartz cell was used for this purpose, and the analysis and manipulation of the data were performed using the UV Probe software version 2.51. The spectral slit width was 2 nm, and the scanning speed was 2800 nm/min. MATLAB^®^ for Windows™ Version 7.0.1 was utilized for all data measurement and processing computations. The PLS toolbox Version 2.1 and iToolbox were also utilized for variable selection and siPLS model construction.

### Chemicals and solvents

Pure SAF (purity of 100.05%±1.436) was provided by EVA Pharma for the Pharmaceutical Industry, (Giza, Egypt). The compound’s purity was measured using the reported HPLC method^25^. The 4-HBD standard with a purity of 99% was supplied by Acros Organics, Fisher Scientific (Belgium).

Parkimedine^®^ Tablets are labelled to contain 100 mg of SAF per tablet and are produced by EVA Pharma for the Pharmaceutical Industry (B.N. (10) 2112425).

Methanol used in this study was HPLC-grade and provided by Fisher scientific (UK). Analytical-grade solvents and reagents were utilized for the stability study. Hydrochloric acid (37%), sodium hydroxide, and hydrogen peroxide (30%) were procured from PioChem Co. (Giza, Egypt).

### Stock standard solution

Stock solutions containing 100.00 µg/mL of SAF and 4-HBD are used throughout the study. Solutions of SAF degradation products were prepared in methanol to achieve final concentrations of 100.0 µg/mL for each degradation product.

For the preparation of the calibration and validation sets, various aliquots were transferred from the four corresponding standard solutions into a series of 10-mL volumetric flasks to prepare solutions with varying ratios of the analyzed components. The volumes were completed with methanol.

### Stress stability studies for SAF

Comprehensive stability investigations were conducted in accordance with ICH guidelines, encompassing hydrolytic (acidic and basic), oxidative, photolytic, and thermal stress conditions^36^. It was found that SAF is likely to undergo acid, base hydrolysis, and oxidative degradation. SAF underwent complete hydrolytic degradation through refluxing with 5.0 N HCl at 100 °C for 5 h or 5.0 N NaOH at 100 °C for 5 h. For the oxidative degradation product, SAF was combined with 30.0 mL of 30.0% H_2_O_2_ at room temperature for 24 h, as shown in Fig. S1. The isolated degradation products were analyzed using MS and IR to ascertain and verify their composition^32^, as illustrated in Fig. S2 and S3.

### Construction of the calibration model

A calibration set was generated by utilizing randomly selected 17 samples with different concentrations of SAF, 4-HBD, SAF hydrolytic degradation, and oxidative degradation products. Mixtures were prepared by transferring and diluting the appropriate volumes for each compound from its working solutions (100.00 µg/mL). The four components’ concentrations in the resulting mixtures were in the range of 3.00 to 23.00, 1.00 to 5.00, 2.50 to 6.50, and 5.00 to 13.00 µg/mL, respectively. These mixtures’ absorption spectra were measured at intervals of 0.1 nm, covering the wavelength range of 220.0–400.0 nm. Calibration models were developed after exporting the recorded spectral data for additional Matlab processing with the PLS and iToolbox tools.

### Validation of calibration models

The developed calibration models were validated with both internal and external validation sets. “Leave one out” was used as a cross-validation for internal validation during the model-building process. Conversely, model prediction and performance assessment were evaluated using an external independent validation set with eight mixtures and four components.

### Application to Parkimedine^®^ tablets

Ten Parkimedine^®^ tablets, each containing 100 mg of SAF, were individually weighed and finely powdered. An amount equivalent to one tablet was accurately transferred to a 100-mL volumetric flask, and 20 mL of methanol was added. The mixture was sonicated for 1 h, then diluted to volume with methanol to obtain a stock solution of 1000 µg/mL, which was subsequently filtered. Appropriate dilutions were prepared to yield a working solution of 100 µg/mL, followed by further dilution with methanol to achieve a final concentration of 6 µg/mL of SAF. The SAF content was quantified using the developed calibration models, and the absorption spectra of the final solution were recorded over the range of 220.0–400.0 nm at 0.1 nm intervals.

## Results and discussion

**Fig. 1:**
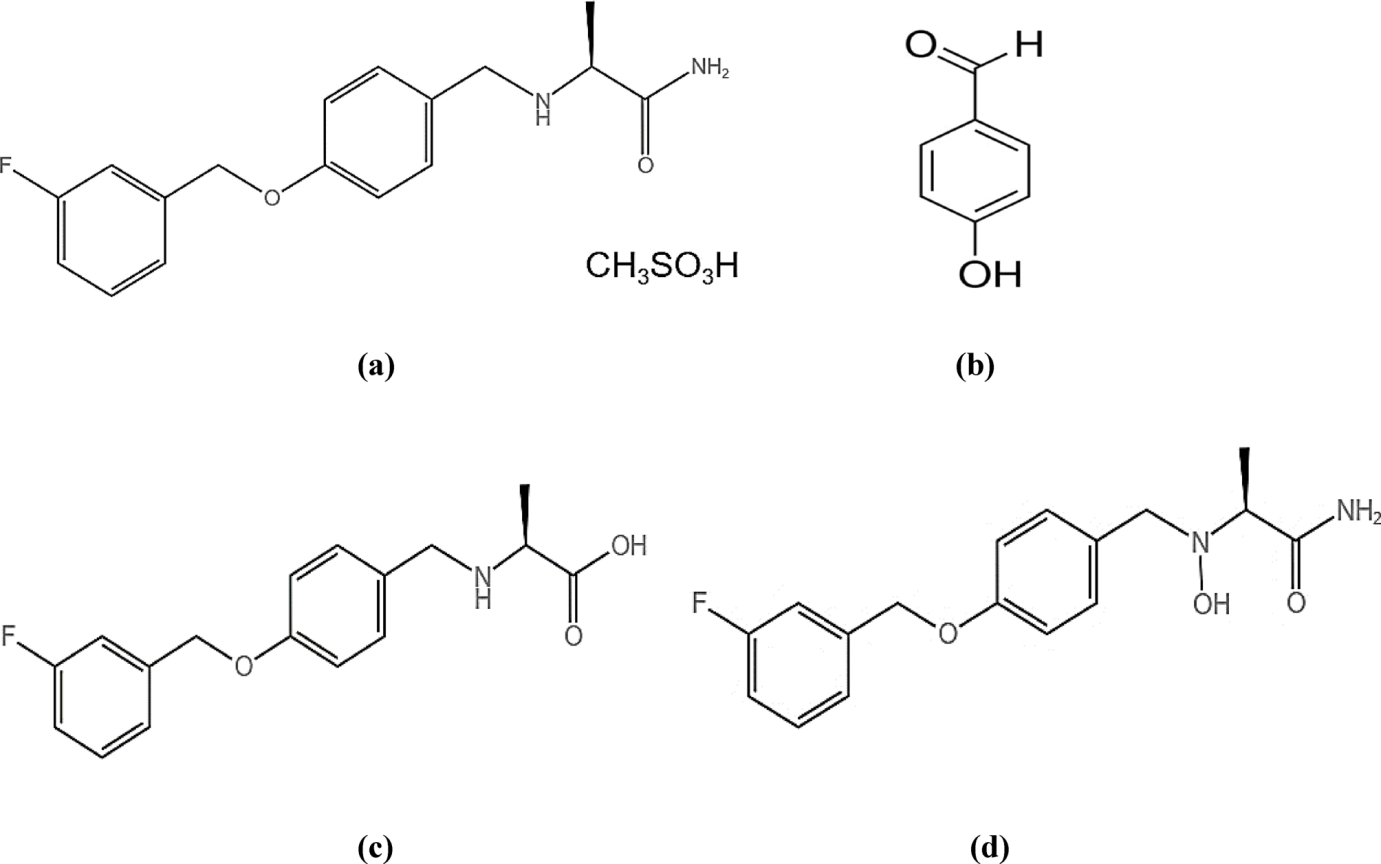
Chemical structure of (**a**) Safinamide mesylate (SAF), (**b**) 4-Hydroxybenzaldehyde (4-HBD), (**c**) SAF hydrolytic degradation product, and (**d**) SAF oxidative degradation product.

**Fig. 2:**
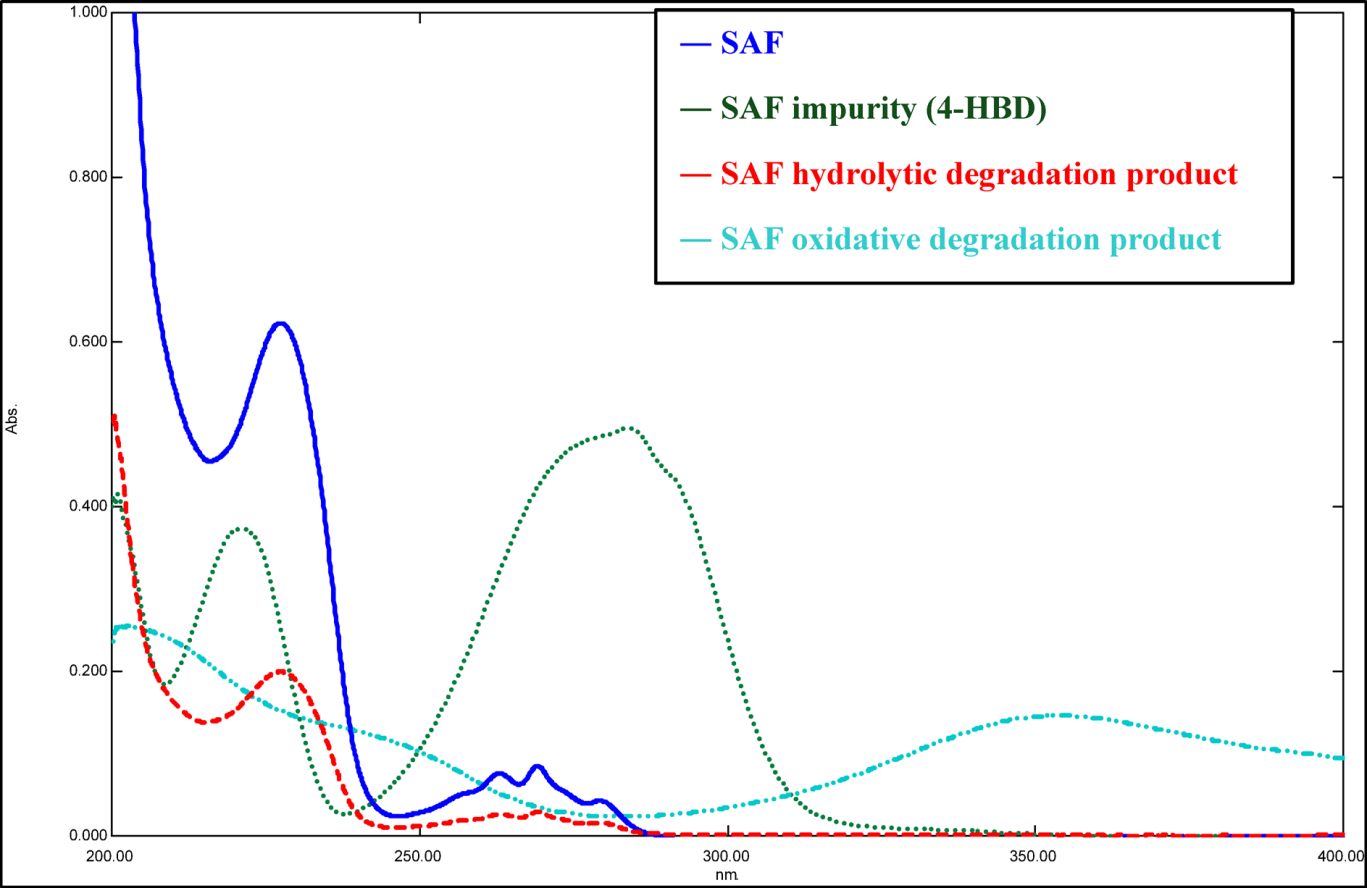
Absorption spectra of 13.00 µg/mL Safinamide mesylate (SAF), 3.00 µg/mL SAF impurity (4-HBD), 4.50 µg/mL SAF hydrolytic degradation product, and 9.00 µg/mL SAF oxidative degradation product using methanol as a blank.

Spectrophotometry is a cost-effective, rapid, and straightforward technique compared to expensive chromatographic methods, enabling precise and accurate analysis of multiple mixtures^37^. Spectrophotometry was then coupled with advanced multivariate techniques to achieve our goal of simplicity, enabling the simultaneous resolution and determination of the cited drug, its synthetic precursor impurity, and its stress-induced degradation products, despite their overlapping spectral signals. Chemometrics is the scientific process of extracting valuable information from analytical numerical data^35,38^. This study involved the development of three different multivariate calibration models; PCR, PLS, and siPLS. PCR and PLS models are often used in quantitative pharmaceutical analysis to extract specific data from broader datasets^39^. Furthermore, advanced chemometric algorithms, including siPLS, have been recently introduced and applied to all numerical data sets. These algorithms utilize signal selection to enhance performance^40^. Nowadays, pharmaceutical research necessitates impurity profiling. It encompasses the sequential isolation, characterization, and quantitative determination of these impurities^41^. Impurities may be present in pharmaceutical formulations due to various factors, including the active pharmaceutical ingredients, inert additives, formulation, and packaging processes^42^. The authors therefore concentrated on determining the drug under investigation; SAF, its synthetic precursor impurity (4-HBD), and its stress-induced degradation products. Figure 1 illustrates the chemical structures of the compounds under study. Figure 2 displays the absorption spectra of the four compounds in methanol as a solvent. Because of the extreme overlap in their spectra, it is not possible to determine the four compounds directly. Univariate spectrophotometric techniques are unable to resolve this kind of overlapping and similarity in spectra. Therefore, Multivariate calibration techniques can be beneficial because the analyzed data can rapidly and accurately resolve and determine each of the four components^38^. A five-level, four-factor calibration model was developed to prepare mixtures of SAF, its synthetic precursor impurity (4-HBD), and its stress-induced degradation products. As shown in Table 1, a training set of 17 mixtures was randomly selected, and an external validation set consisting of the remaining eight mixtures was used to build the regression models. Each spectrum contained 1801 data points, as the scanning range of the prepared samples was 220.0–400.0 nm, and spectral data were acquired at intervals of 0.1 nm. The final spectrum data matrix consists of 1801 columns (25 × 1801), representing the wavelengths, and 25 rows, representing the 25 samples.

**Table 1:** Concentration of the four studied compounds in the calibration and validation sets ^a^.

Samples	SAF	SAF impurity (4-HBD)	SAF Hydrolytic Degradation product	SAF Oxidative Degradation product
1	13	3	4.5	9
2	13	1	2.5	13
3	3	1	6.5	7
4	3	5	3.5	13
5	23	2	6.5	9
6	8	5	4.5	7
7	23	3	3.5	7
8	13	2	3.5	11
**9**	**8**	**2**	**5.5**	**13**
10	8	4	6.5	11
**11**	**18**	**5**	**5.5**	**9**
12	23	4	4.5	13
13	18	3	6.5	13
14	13	5	6.5	5
15	23	5	2.5	11
16	23	1	5.5	5
17	3	4	2.5	9
**18**	**18**	**1**	**4.5**	**11**
**19**	**3**	**3**	**5.5**	**11**
**20**	**13**	**4**	**5.5**	**7**
**21**	**18**	**4**	**3.5**	**5**
**22**	**18**	**2**	**2.5**	**7**
23	8	1	3.5	9
**24**	**3**	**2**	**4.5**	**5**
25	8	3	2.5	5

^a^ The bold samples are those used as an independent (external) validation set.

### Variables selection algorithms

#### Principal component regression (PCR) and partial least squares (PLS)

The multivariate calibration models, such as PCR and PLS, are based on principal component analysis (PCA). These models facilitate the efficient and simplified quantification of multiple analytes across various samples^43^. In the PCR model, PCA is applied to the predictor variables before regression. This approach serves as a powerful exploratory tool for investigating mixtures with uncertain or variable compositions. The components in this model are based on absorbance data without integrating concentration information. Therefore, the resultant components may not achieve optimal predictive accuracy. PLS, on the other hand, combines concentration and absorbance data simultaneously^44^. It then identifies components, known as latent variables (LVs), that maximize the covariance between the two datasets^45^. This integrated methodology typically provides enhanced predictive accuracy, particularly under noisy conditions. As a result, more efficient quantification was achieved using a smaller number of LVs within the spectral range of 220.0–400.0 nm.

**Fig. 3:**
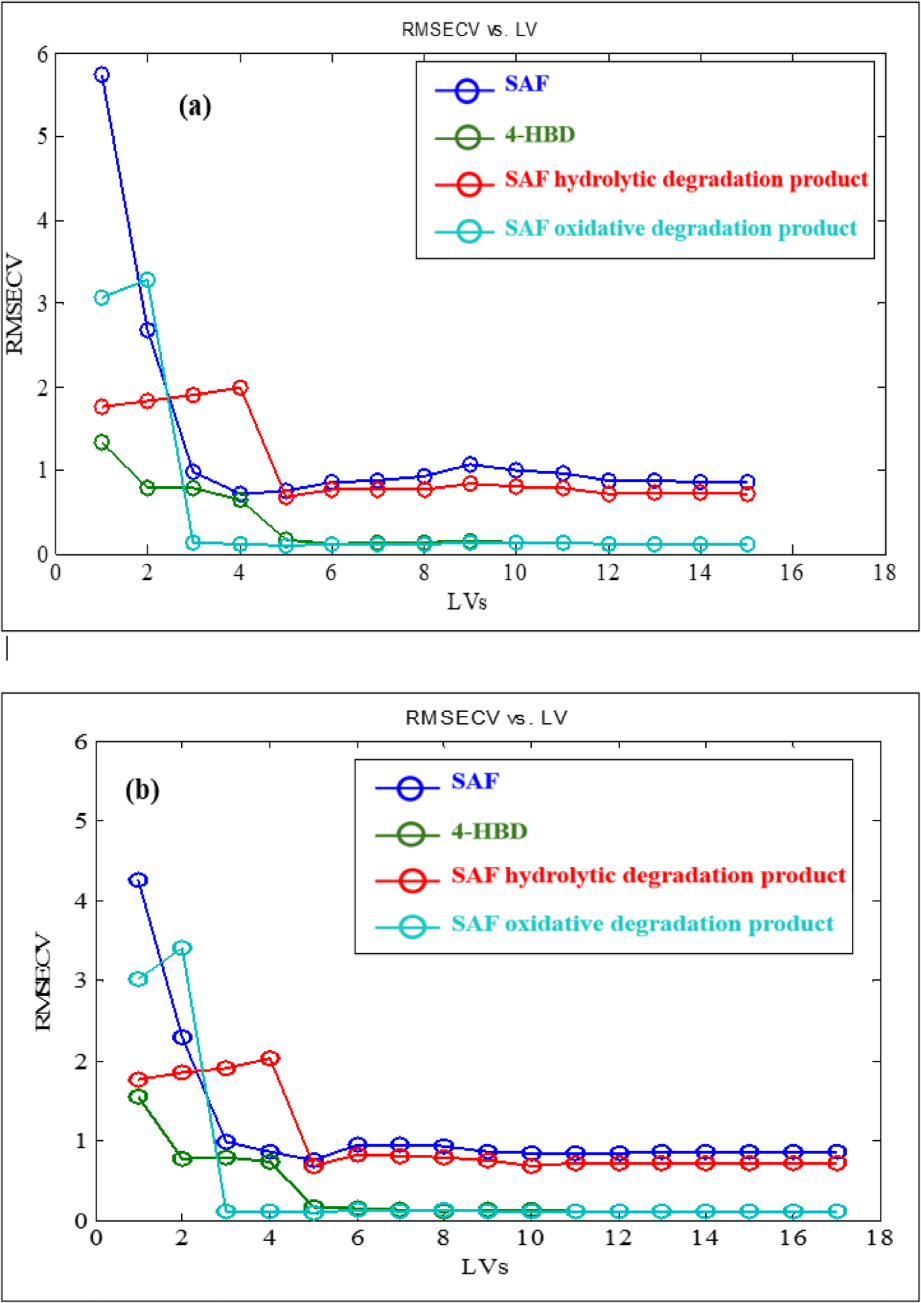
The number of latent variables (LVs) of the developed full spectrum (**a**) PCR and (**b**) PLS models for Safinamide mesylate (SAF), SAF impurity (4-HBD), SAF hydrolytic and oxidative degradation products.

**Fig. 4:**
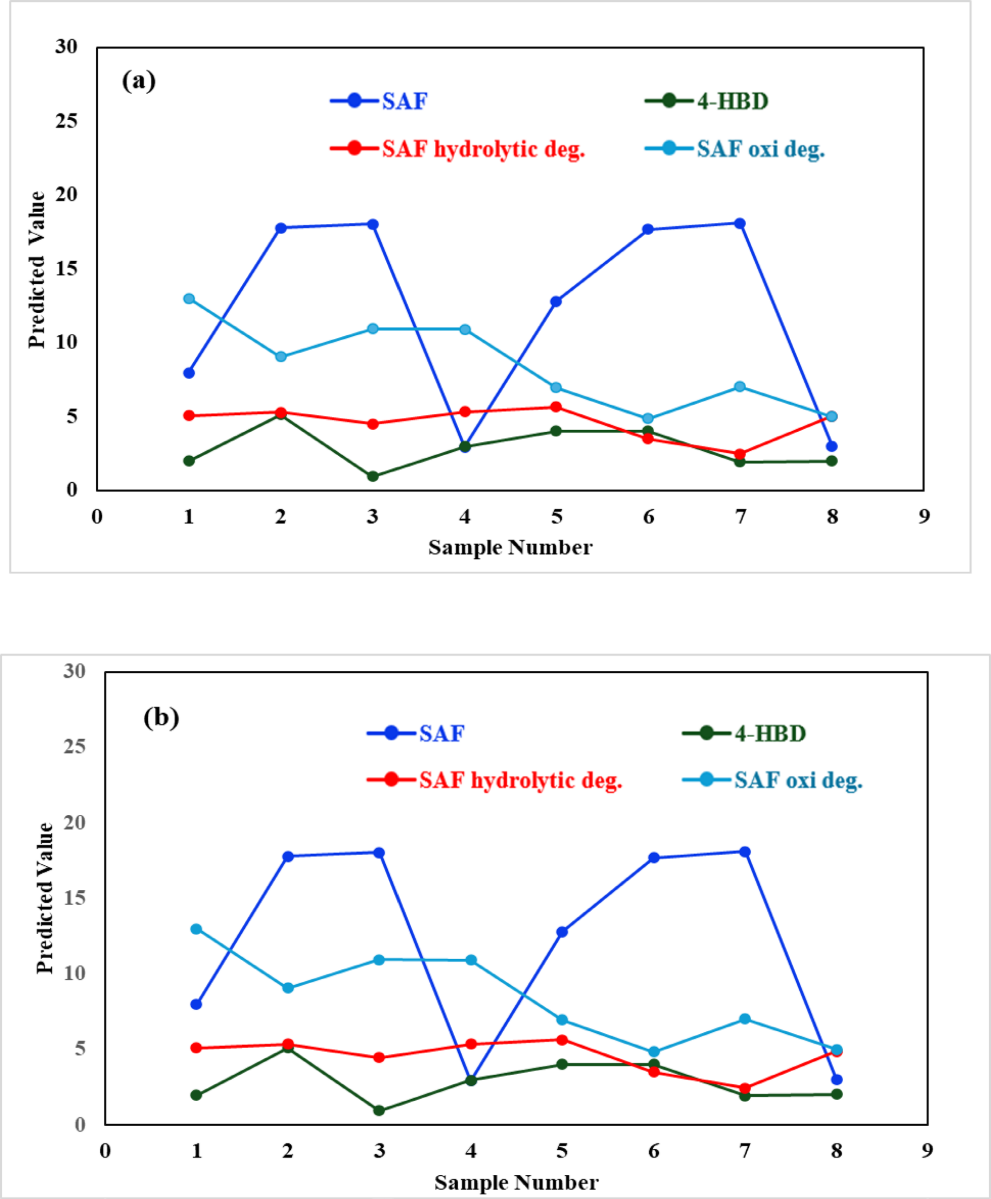
Sample predictions from the validation set for Safinamide mesylate (SAF), SAF impurity (4-HBD), SAF hydrolytic and oxidative degradation products developed by (**a**) PLS and (**b**) PCR models.

The raw data from 17 calibration mixtures underwent auto-scaling and mean centering as a preprocessing step for applying PCR and PLS. However, neither of these preprocessing methods proved to be effective. Various cross-validation methods, including leave-one-out, venetian blinds, contiguous blocks, and random subsets was tried to optimize models construction^46^. The best results were obtained when “leave-one-out” was used as a cross-validation method. The entire data spectrum failed to accurately determine the complex four-component system. Selecting a specific wavelength range can enhance prediction accuracy by identifying the most relevant spectrum band. Consequently, the spectral band from 220.0 to 400.0 nm, with a 0.1 nm interval, is more effective with fewer LVs. The root mean square error of cross validation (RMSECV) were calculated to be 0.6157 and 0.6007 for PCR and PLS, respectively, with five LVs for both models (Fig. 3 and 4).

#### siPLS model

**Fig. 5:**
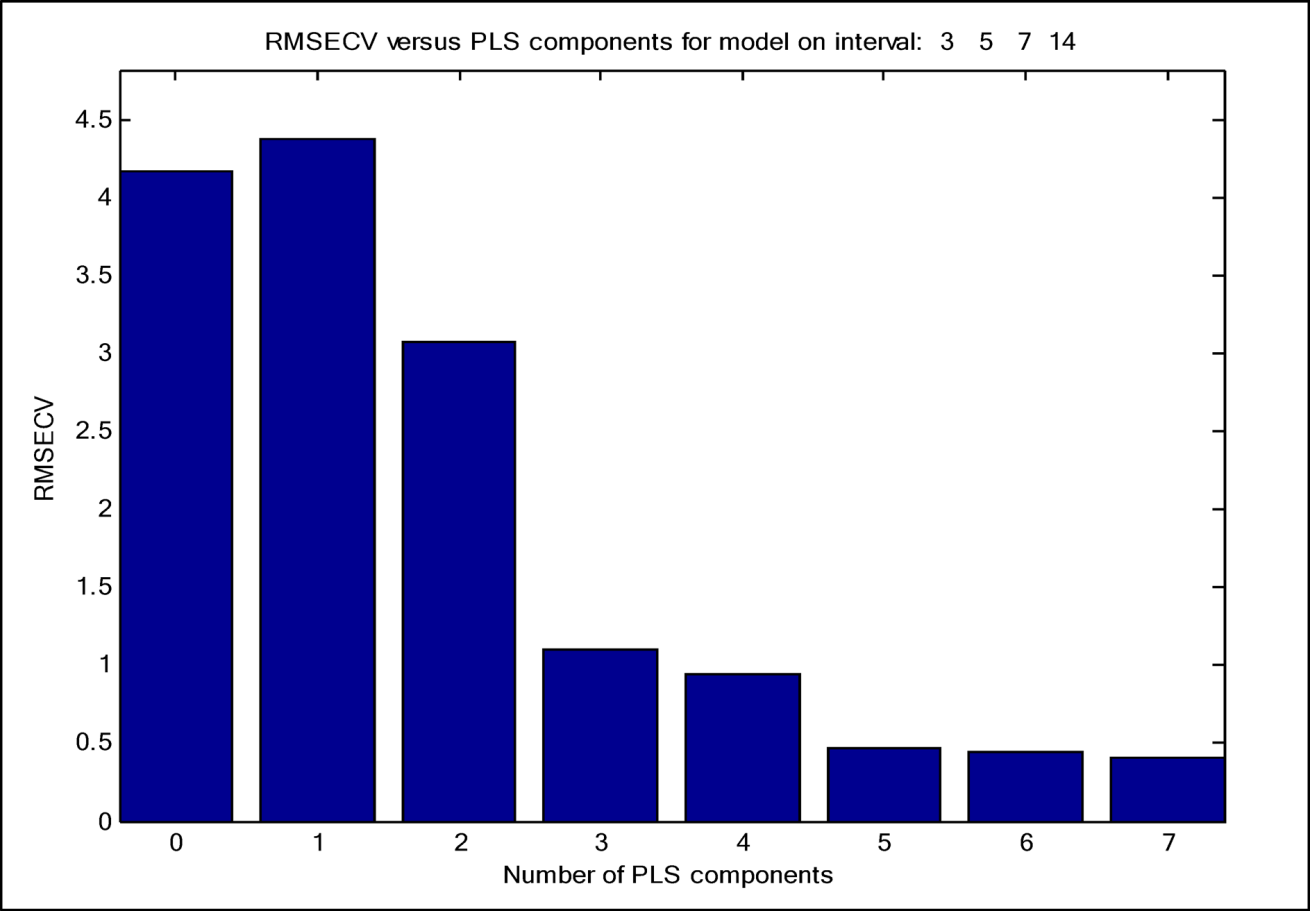
RMSECV against PLS components for siPLS model on interval of [3:5:7:14].

**Fig. 6:**
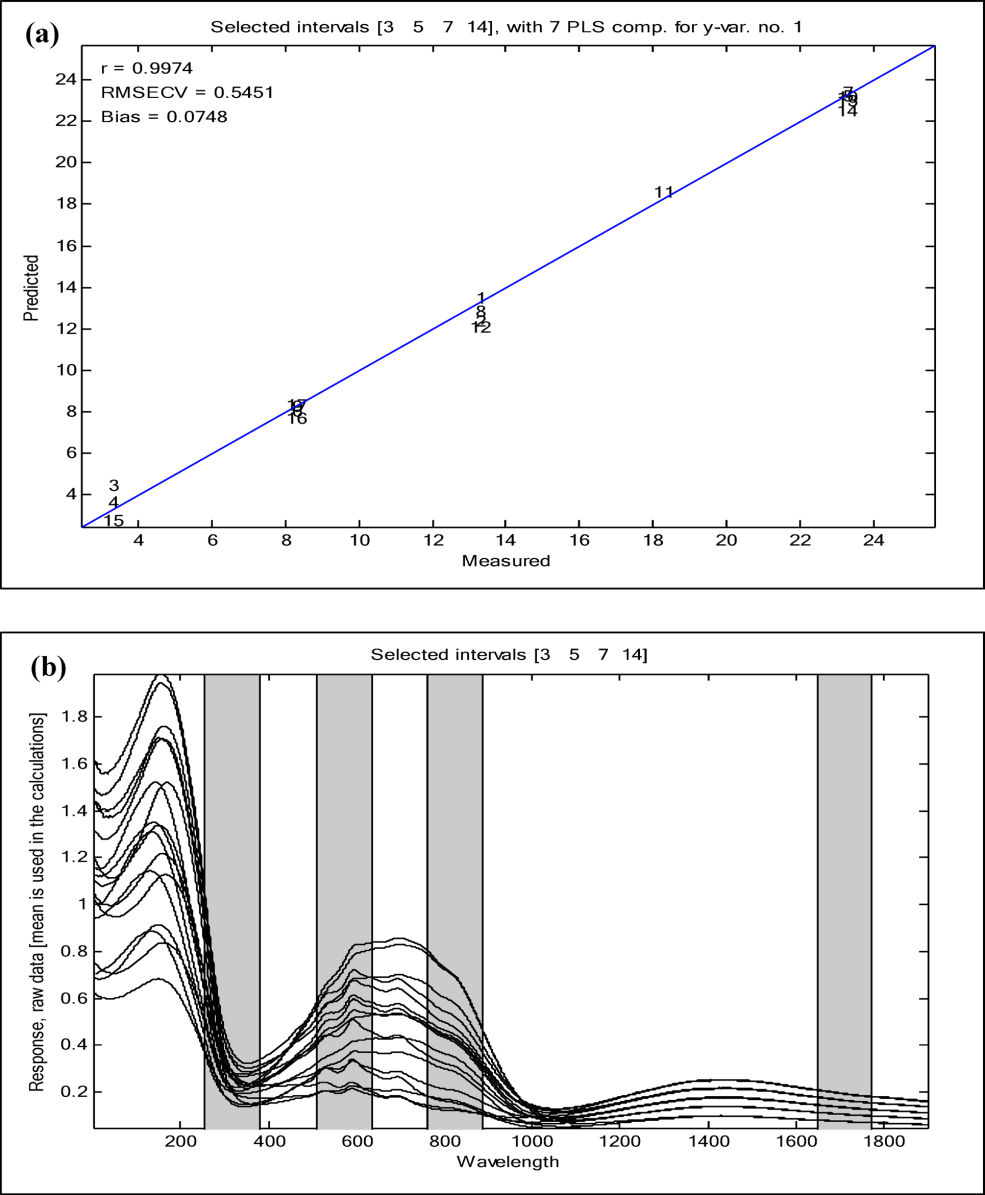
Spectral regions selected to build the models and results: (**a**) siPLS model by combination of subintervals 3, 5, 7, and 14 for quantification; (**b**) average content of the four components (µg/mL) against the predicted values by cross-validation for the siPLS model with 7 LVs.

**Fig. 7:**
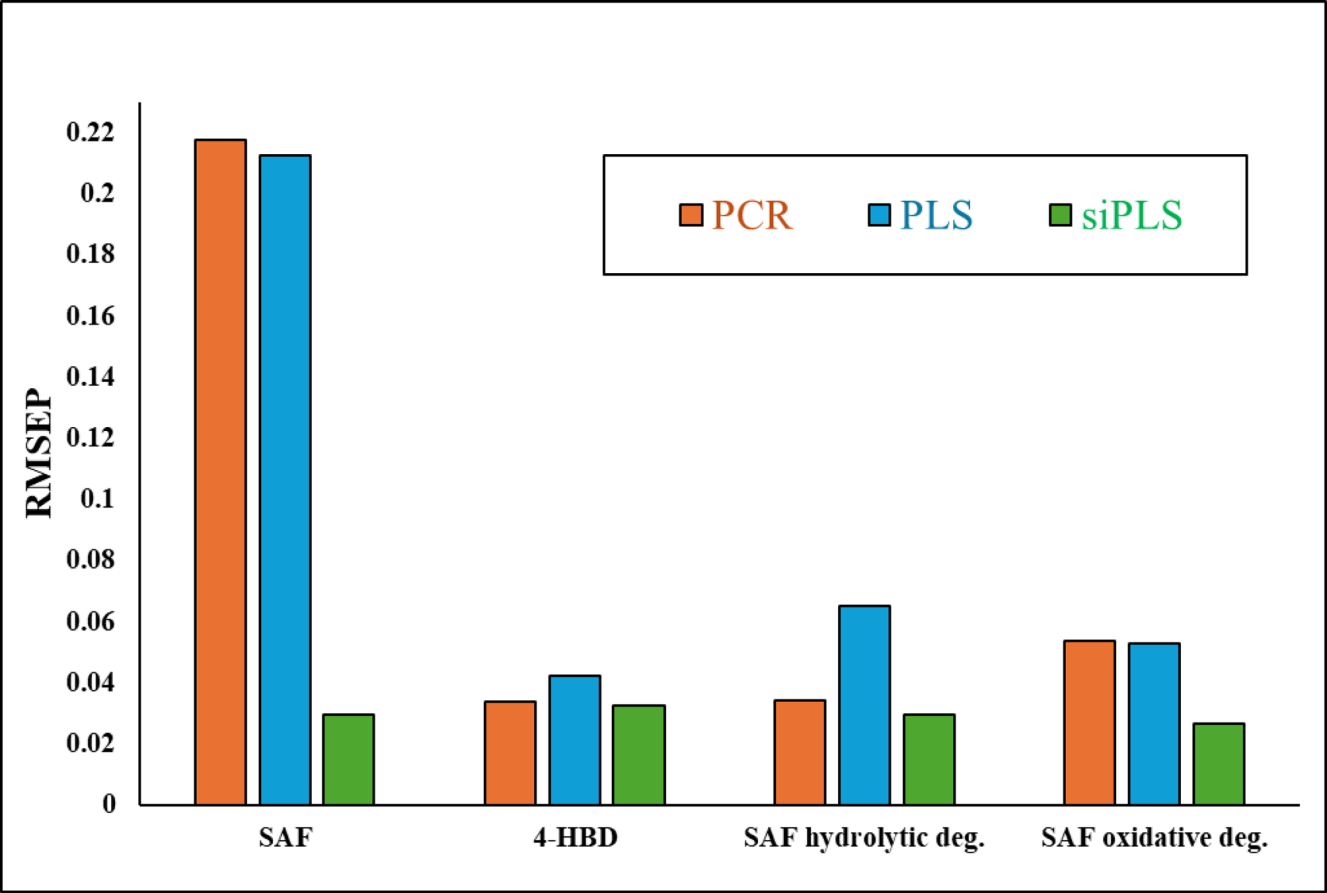
Comparison of RMSEP of Safinamide mesylate (SAF), SAF impurity (4-HBD), SAF hydrolytic degradation product, and SAF oxidative degradation product between the three proposed chemometric methods.

The siPLS model is a variable selection technique designed to optimize a dataset by identifying the most informative spectral regions^47^. The division of the data set into equidistant intervals is the foundation of the siPLS variable selection method, which determines all likely siPLS models by counting the number of selected combined intervals. Subsequently, the results are automatically shown as PLS components’ number and interval combinations. The RMSECV values are computed for the unique models. It is important to note that these values are primarily influenced by the number of intervals and the combinations of intervals. The siPLS model was utilized to identify the most informative regions in the studied mixture, resulting in improved prediction ability for the components, reduced interference, and a decrease in the number of LVs compared to PCR and PLS. A variety of equidistant interval combinations were created and examined. PLS regression model was used for all combinations of two, three, and four intervals. Figures 5 and 6 illustrate the best combination of four subintervals [3:5:7:14] and seven LVs with RMSECV value of 0.5451and RMSE value of 0.4077, as illustrated in Table S1. In order to demonstrate the superiority of siPLS over the implemented PCR and PLS models, Fig. 7 compares the RMSEP values of the three models for the four studied components.

### Method validation

The validation of analytical methods encompasses multiple aspects. For numerical validation, data is typically split into calibration and test sets. It is essential to ensure that the objects in the data are not stratified in a way that could bias the model’s findings. This may impact the interpretation in terms of the model’s dimensionality, the identification of significant variables, and the understanding of the actual correlations among the variables within the system. Validation is essential to prevent inaccurate estimations regarding a regression model’s ability to classify new samples or measure the dependent variables of interest. While it is widely believed that a pure, independent test set is always required for validation, the calibration set validation also needs to consider any subgroups of objects that explain the uncontrolled variance, which is unknown for future samples. Validation is a crucial process that aims to provide answers to important questions. It is essential to carefully consider the nature of the question or hypotheses being tested in order to choose the most suitable validation strategy. Recently, concerns have arisen over impurities in the assay of active pharmaceutical ingredients (APIs). Most APIs have probable impurities; these impurities have physical and chemical properties similar to those of pharmaceutical substances because they share a similar structure. Resolving and quantifying APIs in the presence of impurities with identical structures and spectra can pose challenges during method development and validation. Our study aims to determine the SAF, its synthetic precursor impurity (4-HBD), and stress-induced degradation products^14^. The direct detection of the four compounds is difficult due to the overlap of their spectral signals, as demonstrated in Fig. 2. Univariate calibration methods are incapable of resolving this overlap and spectral similarity. This is due to the fact that multivariate calibration is advantageous in that it allows for the resolution and efficient determination of each of the four components by subjecting a substantial amount of information about these intricate systems to an algorithm. The regression models were developed using 17 mixtures as a training set, and 8 mixtures were used for external validation. In this four-factor calibration design, the concentration of each component must be orthogonal to the concentrations of the other components in the mixtures (Table 2).

**Table 2:** Statistical and linear regression parameters for the validation set using PCR, PLS and SiPLS models.

Parameters	PCR				PLS				siPLS [ 3 5 7 14]			
	SAF	SAF impurity	SAF hydrolytic Deg.	SAF Oxidative Deg.	SAF	SAF impurity	SAF hydrolytic Deg.	SAF Oxidative Deg.	SAF	SAF impurity	SAF Hydrolytic Deg.	SAF Oxidative Deg.
Mean recovery %	99.35	99.67	100.02	99.68	99.39	99.99	99.68	99.69	100.26	100.39	100.27	99.80
SD	1.067	2.020	5.791	1.089	1.246	1.744	4.695	1.092	0.844	1.678	1.195	1.033
RSD %	1.074	2.027	5.790	1.093	1.254	1.744	4.710	1.096	0.842	1.671	1.192	1.035
Correlation coefficient (r)	0.9998	0.9998	0.9678	0.9998	0.9998	0.9997	0.9785	0.9998	0.9999	0.9996	0.9986	0.9998
Slope	0.9947	1.0376	0.9324	1.0030	0.9947	1.0291	0.9498	1.0025	0.9983	1.0008	0.9913	1.0056
Intercept	-0.0113	-0.0961	0.3003	-0.0442	-0.0101	-0.0686	0.2090	-0.0402	0.0317	0.0091	0.0507	-0.0569
LOD ^a^	0.468	0.093	0.967	0.212	0.482	0.106	0.783	0.213	0.340	0.120	0.199	0.195
LOQ ^a^	1.418	0.281	2.930	0.644	1.459	0.322	2.374	0.647	1.032	0.363	0.603	0.590

^a^ Calculated from equation [LOD (limit of detection) = 3.3 (SD/S), LOQ (limit of quantification) = 10 (SD/S); where SD is the standard deviation of regression residuals and S is the slope of the calibration curves.

### Application to Parkimedine^®^ tablets

The suggested PCR, PLS, and siPLS models were suitable and valid for analyzing SAF in Parkimedine^®^ tablets. Further evidence for the validity of the presented methods is provided using the standard addition technique. The results, as shown in Table 3, demonstrate that when the suggested procedures were followed, excipients did not affect the analysis. Moreover, the determination results were discovered to be quite similar, suggesting a remarkable coincidence between the label amounts and the determination results, as shown in Table S2.

**Table 3:** Determination of Safinamide mesylate (SAF) in Parkimedine^®^ tablets using the proposed chemometric methods and the results of standard addition technique.

Pharmaceutical formulation	Proposed chemometric methods				Standard addition technique	
	Drug	Models	Claimed (µg/mL)	%Found ± SD^a^	Pure added (µg/mL)	%Recovery of the pure added^b^
Parkimedine^®^ tablets(Each tablet was Labelled to Contain 100 mg SAF)	SAF	PCR	6	98.55 ± 0.987	3.0	98.67
					6.0	100.61
					12.0	102.05
					Mean ± SD	100.44 ± 1.692
		PLS		98.92 ± 0.947	3.0	99.66
					6.0	100.90
					12.0	102.15
					Mean ± SD	100.90 ± 1.245
		siPLS		100.33 ± 0.722	3.0	101.99
					6.0	102.05
					12.0	99.13
					Mean ± SD	101.06 ± 1.664

^a^ Average of five determinations.

^b^ Average of three experiments.

### Sustainability assessment

Analytical techniques should prioritize environmental sustainability. This can be achieved by improving analytical procedures, eliminating or reducing the use of hazardous reagents, conserving energy, and enhancing safety for analysts. Sustainability should be prioritized at every stage of the analysis process, from sample collection to the management of analytical waste^48^. As such, it is crucial to evaluate the impact of analytical techniques on the environment and potential workforce consequences, particularly when assessing their eco-friendliness. Various evaluation techniques have been developed to assess the sustainability of analytical processes^49^.

#### Complex modified GAPI (ComplexMoGAPI) approach

**Fig. 8:**
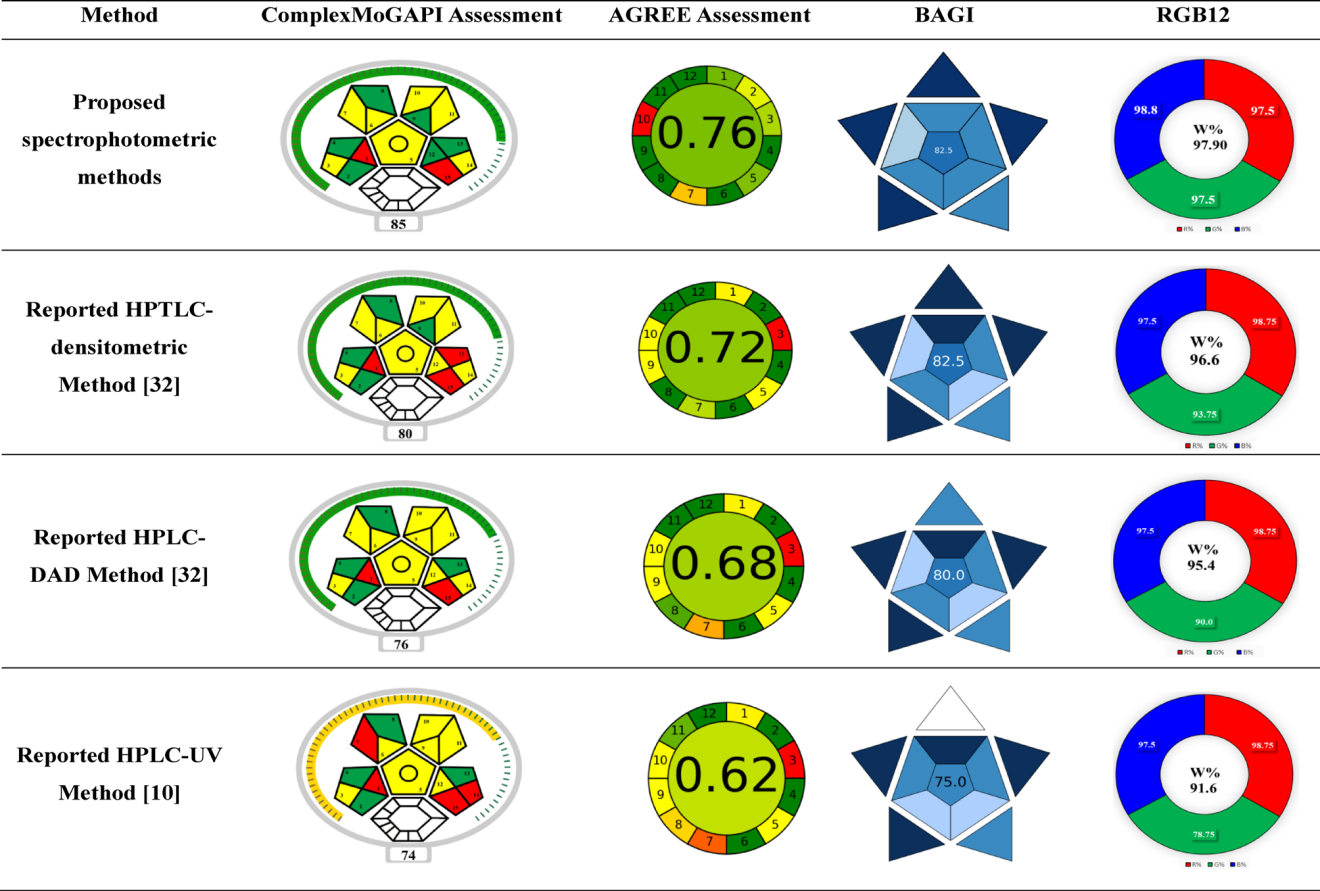
Greenness assessment of the proposed spectrophotometric methods and the reported ones according to ComplexMoGAPI, AGREE tools, blueness assessment by BAGI tool and whiteness assessment by RGB 12 model.

This tool introduces a modified version of Complex-GAPI called ComplexMoGAPI. It combines the attractive visuals of Complex-GAPI with accurate total scores. ComplexMoGAPI provides a comprehensive evaluation of the eco-friendliness of methods, enabling more precise and impartial method comparisons^50^. The software provided enhances the application process, streamlining assessments for a more efficient and user-friendly experience. MoGAPI provides a variety of options beyond the conventional red/green/yellow icons that are present in Complex-GAPI. It generates a cumulative score to assess the method’s overall environmental sustainability. The scoring system takes into account the variety of options available in each category^50^. Figure 8 demonstrates the favorable results obtained from the application of the suggested techniques compared to the reported methods^10,32^, evidenced by an increase in green-shaded sections and a decrease in red-shaded sections.

#### Greenness assessment applying analytical GREEness metric (AGREE) approach

The overall greenness of the analytical methodology is evaluated using the AGREE tool. The software produces an array of colors representing the degree of compliance with the twelve fundamentals of the GAC, which is then converted into a score ranging from zero to one^51^. It is available for free download. A circular pictogram with 12 sections is part of the final assessment report. The width can be adjusted according to the importance of each section. The evaluation of greenness varied from low (= 0) to high (= 1) using a color scale ranging from deep red to deep green^51^. The established chemometric approaches produced an AGREE pictogram with an overall score (0.76) higher than that reported in the methods^10,32^, as shown in Fig. 8.

#### Blue applicability grade index (BAGI)

The BAGI tool is an innovative measure for assessing the practicality of an analytical method. It focuses on applying White Analytical Chemistry and can be seen as an extension of current green metrics^52^. The BAGI metric tool yields two distinct sets of results: a numerical score at the center and a pictogram of an asteroid, serving as a visual representation. The asteroid’s pictogram shows different levels of compliance using a range of blue colors: light blue for low compliance, dark blue for high compliance, blue for moderate compliance, and white for non-compliance. This pictogram graphically shows the evaluation result. This pictogram graphically shows the evaluation result. The established chemometric approaches have a higher BAGI score than the reported ones^10,32^, indicating their good applicability, as shown in Fig. 8.

#### Whiteness tool for assessing analytical chemistry method

The WAC metric tool aims to evaluate the economic viability, environmental impact, and efficiency of analytical techniques. There are several methods to evaluate sustainability, each with its own advantages and disadvantages. However, integrating different approaches consistently yields positive results^53^. The Red-Green-Blue (RGB) model is widely used to assess analytical procedures globally, employing a quantitative approach to evaluate their impact on sustainability^54,55^. The whiteness score quantifies the adherence of a method to WAC principles using the RGB 12 model, which evaluates three aspects: red (efficiency and validity), green (safety, waste, and energy), and blue (economics, time, implementation, and practicality). The analysis yields a numerical score from 0 to 100, showing the overall sustainability of the analytical method. Based on the data in Fig. 8, the proposed chemometrics methods are more environmentally friendly than the reported ones^10,32^.

### Statistical comparison

The results for the analysis of SAF in pharmaceutical dosage by applying the proposed methods and were compared to those obtained by applying reported HPLC method^32^. Results from the PCR, PLS, and siPLS methods did not significantly differ from the reported method in terms of accuracy and precision as determined by Student’s t-test and variance ratio F-test, respectively. Table S3 shows the results.

## Conclusion

In our study, we employed green, simple, rapid, and practical data analysis techniques, including PCR, PLS, and siPLS models, to address the issue of SAF spectrum similarity and high overlapping with its synthetic precursor impurity (4-HBD) and stress-induced degradation products. The development of spectrophotometric methods has proven to be a practical option for rapid mixture analysis, as it eliminates the need for costly and time-consuming separation techniques. The well-established chemometric models can be utilized in quality control laboratories for regular analysis of pure SAF or its pharmaceutical formulations free from impurities and excipients. There is no need for complex equipment, sensitive reactions, or pre-separation procedures for these substances. The methods also function in laboratories without readily available liquid chromatographic equipment. Compared with the PCR and PLS models, the siPLS model exhibited a lower mean square error and superior performance in quantifying SAF in its pharmaceutical formulation, even in the presence of its precursor impurity (4-HBD) and stress-induced degradation products in synthetic mixtures. Future research could expand the application of the developed chemometric models to more complex sample matrices or additional multicomponent systems. The potential of these models to be utilized for point-of-care diagnostics and on-site analytical testing could be enhanced by incorporating them into miniaturized or portable spectroscopic platforms. Moreover, they would be easier to implement in industrial quality control settings after comprehensive validation in line with GLP and GMP standards.

## Supplementary Information

Below is the link to the electronic supplementary material.

Supplementary Material 1

## Data Availability

All data generated or analyzed during this study are included in this published article.
