# Supplementary material for: Chemometric-assisted spectrophotometric approach for stability assessment of safinamide and its synthetic precursor in antiparkinsonian formulation with sustainability profiling
Source: Sci Rep. 2025 Nov 29;15:42925. doi: 10.1038/s41598-025-28085-4 (PMC12672687; doi:10.1038/s41598-025-28085-4)
Supplement: Supplementary file 1 — Supplementary Material 1 [file 41598_2025_28085_MOESM1_ESM.docx]

**Supplementary material**

**For**

**Chemometric-assisted spectrophotometric approach for stability assessment of safinamide and its synthetic precursor in antiparkinsonian formulation with sustainability profiling**

Engy A. Ibrahim ^1^, Samah S. Saad ^1^, Maha A. Hegazy ^2^, Laila E. Abdel Fattah ^1^, Hoda M. Marzouk ^3*^

^1^ Pharmaceutical Analytical Chemistry Department, College of Pharmaceutical Sciences and Drug Manufacturing, Misr University for Science & Technology, 6^th^ of October City, Giza, Egypt.

^2^ Pharmaceutical Chemistry Department, Faculty of Pharmacy, Future University in Egypt, Cairo 11835, Egypt

^3^ Pharmaceutical Analytical Chemistry Department, Faculty of Pharmacy, Cairo University, Kasr Al-Aini Street, 11562, Cairo, Egypt

**Table S1**. Statistical results of siPLS model for the four components studied.

| PLS component | Intervals ^a^ | RMSE |
| --- | --- | --- |
| 7 | [ 3 4 6 7] | 0.3645 |
| 7 | [ 3 4 5 7] | 0.3667 |
| 7 | [ 3 5 6 7] | 0.3701 |
| 7 | [ 2 3 6 14] | 0.3954 |
| 7 | [ 2 3 4 5] | 0.3974 |
| 7 | [ 3 4 5 6] | 0.4045 |
| 7 | [ 2 3 6 15] | 0.4050 |
| 7 | [ 2 3 6 13] | 0.4059 |
| 7 | [ 3 4 6 12] | 0.4064 |
| 7 ^b^ | [ 3 5 7 14] | 0.4077 |

^a^ Original number of intervals is 15.

^b^ The gray shade represents the selected interval.

**Table S2.** Determination of Safinamide mesylate (SAF) and its impurities in the validation set by the proposed methods.

| **SAF oxidative degradation product** | | | **SAF hydrolytic degradation product** | | | **SAF impurity (4-HBD)** | | | **SAF** | | | **Mix No.** | **Method** | | |
| --- | --- | --- | --- | --- | --- | --- | --- | --- | --- | --- | --- | --- | --- | --- | --- |
| **% R** | **Found ^a^**  **(μg/mL)** | **Taken**  **(μg/mL)** | **% R** | **Found ^a^**  **(μg/mL)** | **Taken**  **(μg/mL)** | **% R** | **Found ^b^**  **(μg/mL)** | **Taken**  **(μg/mL)** | **% R** | **Found ^a^**  **(μg/mL)** | **Taken**  **(μg/mL)** |  |  |  |  |
| 100.06 | 13.01 | 13.00 | 92.43 | 5.08 | 5.50 | 99.67 | 1.99 | 2.00 | 99.90 | 7.99 | 8.00 | 1 | **PCR** | | |
| 100.74 | 9.07 | 9.00 | 96.36 | 5.30 | 5.50 | 102.77 | 5.14 | 5.00 | 98.88 | 17.80 | 18.00 | 2 |  |  |  |
| 99.52 | 10.95 | 11.00 | 100.14 | 4.51 | 4.50 | 96.24 | 0.96 | 1.00 | 100.28 | 18.05 | 18.00 | 3 |  |  |  |
| 99.42 | 10.94 | 11.00 | 96.78 | 5.32 | 5.50 | 99.69 | 2.99 | 3.00 | 97.95 | 2.94 | 3.00 | 4 |  |  |  |
| 99.57 | 6.97 | 7.00 | 102.88 | 5.66 | 5.50 | 100.51 | 4.02 | 4.00 | 98.52 | 12.81 | 13.00 | 5 |  |  |  |
| 97.29 | 4.86 | 5.00 | 100.00 | 3.50 | 3.50 | 101.03 | 4.04 | 4.00 | 98.23 | 17.68 | 18.00 | 6 |  |  |  |
| 100.58 | 7.04 | 7.00 | 99.54 | 2.49 | 2.50 | 97.54 | 1.95 | 2.00 | 100.56 | 18.10 | 18.00 | 7 |  |  |  |
| 100.30 | 5.01 | 5.00 | 112.06 | 5.04 | 4.50 | 99.90 | 2.00 | 2.00 | 100.46 | 3.01 | 3.00 | 8 |  |  |  |
| **99.68** | | | **100.02** | | | **99.67** | | | **99.35** | | | **% Mean** | | | |
| **0.0538** | | | **0.0344** | | | **0.0338** | | | **0.2177** | | | **RMSEP** | | | |
| **% R** | **Found ^a^**  **(μg/mL)** | **Taken**  **(μg/mL)** | **% R** | **Found ^a^**  **(μg/mL)** | **Taken**  **(μg/mL)** | **% R** | **Found ^b^**  **(μg/mL)** | **Taken**  **(μg/mL)** | **% R** | **Found ^a^**  **(μg/mL)** | **Taken**  **(μg/mL)** | **Mix No.** | | **PLS** | |
| 100.05 | 13.01 | 13.00 | 92.69 | 5.10 | 5.50 | 99.63 | 1.99 | 2.00 | 99.81 | 7.98 | 8.00 | 1 | |  |  |
| 100.72 | 9.06 | 9.00 | 97.63 | 5.37 | 5.50 | 102.35 | 5.12 | 5.00 | 98.82 | 17.79 | 18.00 | 2 | |  |  |
| 99.53 | 10.95 | 11.00 | 99.48 | 4.48 | 4.50 | 97.20 | 0.97 | 1.00 | 100.30 | 18.06 | 18.00 | 3 | |  |  |
| 99.40 | 10.93 | 11.00 | 97.36 | 5.35 | 5.50 | 99.48 | 2.98 | 3.00 | 97.63 | 2.93 | 3.00 | 4 | |  |  |
| 99.58 | 6.97 | 7.00 | 102.58 | 5.64 | 5.50 | 100.67 | 4.03 | 4.00 | 98.52 | 12.81 | 13.00 | 5 | |  |  |
| 97.30 | 4.86 | 5.00 | 100.51 | 3.52 | 3.50 | 100.76 | 4.03 | 4.00 | 98.27 | 17.69 | 18.00 | 6 | |  |  |
| 100.61 | 7.04 | 7.00 | 98.30 | 2.46 | 2.50 | 97.73 | 1.95 | 2.00 | 100.63 | 18.11 | 18.00 | 7 | |  |  |
| 100.37 | 5.02 | 5.00 | 108.89 | 4.90 | 4.50 | 102.09 | 2.04 | 2.00 | 101.10 | 3.03 | 3.00 | 8 | |  |  |
| **99.69** | | | **99.68** | | | **99.99** | | | **99.39** | | | **% Mean** | | | |
| **0.0529** | | | **0.0650** | | | **0.0425** | | | **0.2124** | | | **RMSEP** | | | |
| **% R** | **Found ^a^**  **(μg/mL)** | **Taken**  **(μg/mL)** | **% R** | **Found ^a^**  **(μg/mL)** | **Taken**  **(μg/mL)** | **% R** | **Found ^b^**  **(μg/mL)** | **Taken**  **(μg/mL)** | **% R** | **Found ^a^**  **(μg/mL)** | **Taken**  **(μg/mL)** | **Mix No.** | | | **siPLS** |
| 100.27 | 13.04 | 13.00 | 97.69 | 5.37 | 5.50 | 101.69 | 2.03 | 2.00 | 101.42 | 8.11 | 8.00 | 1 | | |  |
| 100.49 | 9.04 | 9.00 | 100.78 | 5.54 | 5.50 | 101.11 | 5.06 | 5.00 | 99.49 | 17.91 | 18.00 | 2 | | |  |
| 99.71 | 10.97 | 11.00 | 100.39 | 4.52 | 4.50 | 97.70 | 0.98 | 1.00 | 100.73 | 18.13 | 18.00 | 3 | | |  |
| 99.74 | 10.97 | 11.00 | 100.80 | 5.54 | 5.50 | 98.97 | 2.97 | 3.00 | 100.95 | 3.03 | 3.00 | 4 | | |  |
| 99.05 | 6.93 | 7.00 | 100.09 | 5.50 | 5.50 | 99.54 | 3.98 | 4.00 | 98.92 | 12.86 | 13.00 | 5 | | |  |
| 97.68 | 4.88 | 5.00 | 101.32 | 3.55 | 3.50 | 99.62 | 3.98 | 4.00 | 99.59 | 17.93 | 18.00 | 6 | | |  |
| 100.85 | 7.06 | 7.00 | 99.70 | 2.49 | 2.50 | 102.25 | 2.05 | 2.00 | 100.59 | 18.11 | 18.00 | 7 | | |  |
| 100.57 | 5.03 | 5.00 | 101.41 | 4.56 | 4.50 | 102.26 | 2.05 | 2.00 | 100.36 | 3.01 | 3.00 | 8 | | |  |
| **99.80** | | | **100.27** | | | **100.39** | | | **100.26** | | | **% Mean** | | | |
| **0.0267** | | | **0.0297** | | | **0.0324** | | | **0.0297** | | | **RMSEP** | | | |

^a^ Average of three determinations.

**Table S3.** Statistical comparison of the results obtained by the proposed chemometric methods and the reported method for determination of Safinamide mesylate (SAF) in Parkimedine^®^ tablets.

| Parameters | Proposed chemometric methods | | | Reported method ^a)^ |
| --- | --- | --- | --- | --- |
|  | PCR | PLS | siPLS |  |
|  | **SAF** | | | **SAF** |
| Mean | 98.55 | 98.92 | 100.33 | 99.63 |
| SD | 0.987 | 0.947 | 0.722 | 0.523 |
| n | 5 | 5 | 5 | 5 |
| Variance | 0.974 | 0.897 | 0.521 | 0.274 |
| Student’s t-test  (2.306) ^b)^ | 2.16 | 1.47 | 1.756 | ------- |
| F value  (6.39) ^b)^ | 3.55 | 3.27 | 1.90 | ------- |

1. HPLC method using Zorbax C_18_ (150.0 × 4.6 mm, 5.0 μm) as analytical column, mobile phase consists of a mixture of f 25.0 mM sodium dihydrogen phosphate buffer containing 0.1% (w/v) octane sulfonic acid sodium salt adjusted with *o*-phosphoric acid to pH 5.0 ± 0.1 and methanol (45:55, v/v) kept at 40 ◦C. By adjusting the flow rate to 1.2 mL/min and setting it to 1.5 mL/min at 3.1 min at 225.0 nm [32].
2. Tabulated t- and F values at P = 0.05.

**
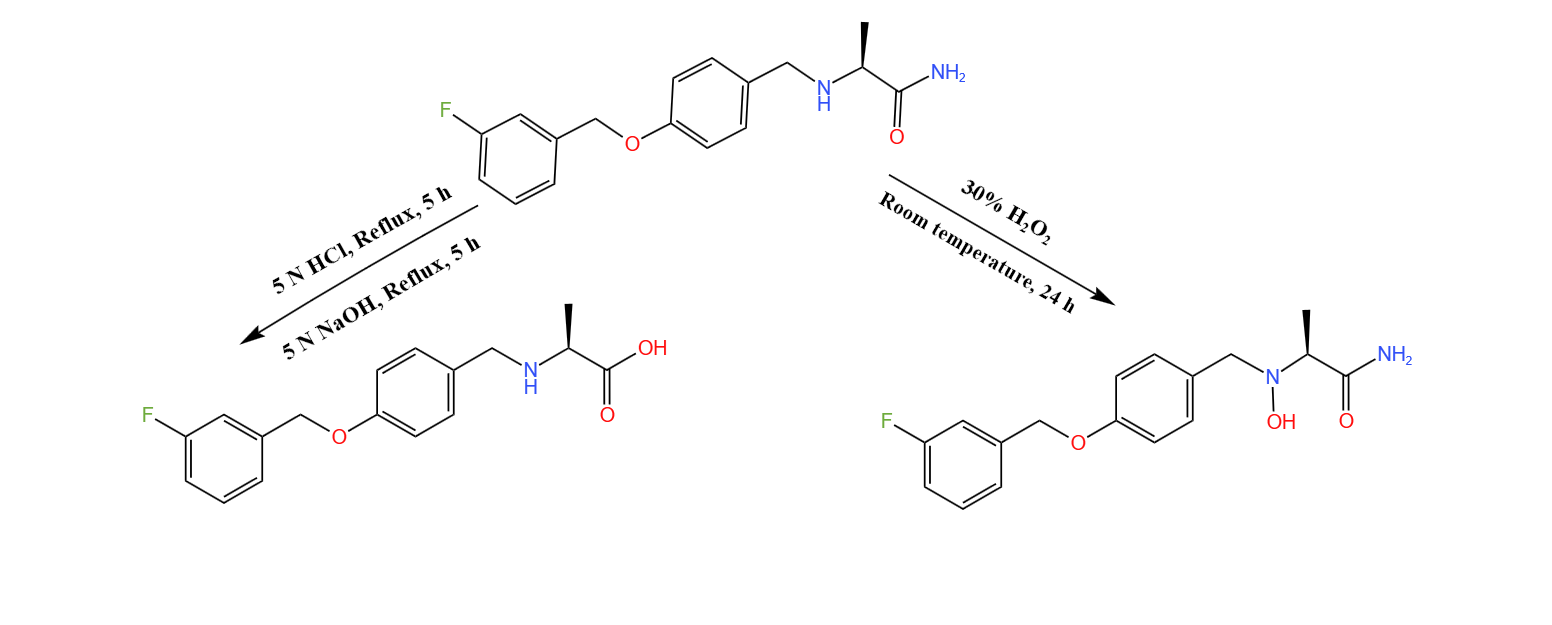
**

Safinamide mesylate (SAF)

SAF Hydrolytic Degradation product

SAF Oxidative Degradation product

**Fig S1.** The suggested pathways for Safinamide-induced degradation.


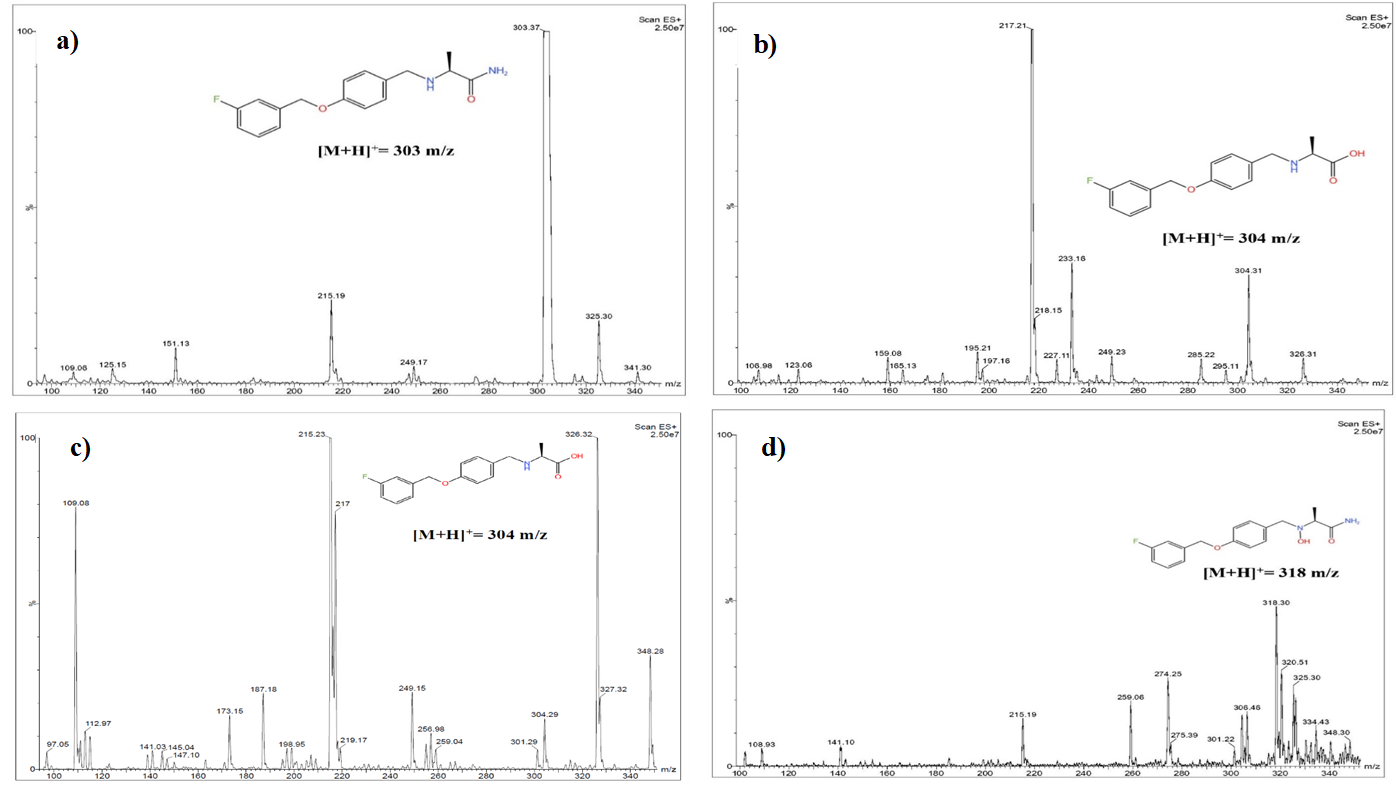


**Fig. S2.** The mass spectrum shows molecular ion peaks at (a) 303.0 m/z for SAF intact drug, (b) 304.0 m/z for acid-induced degradation product, (c) 304.0 m/z for basic-induced degradation, and (d) 318.0 m/z for oxidative degradation product.


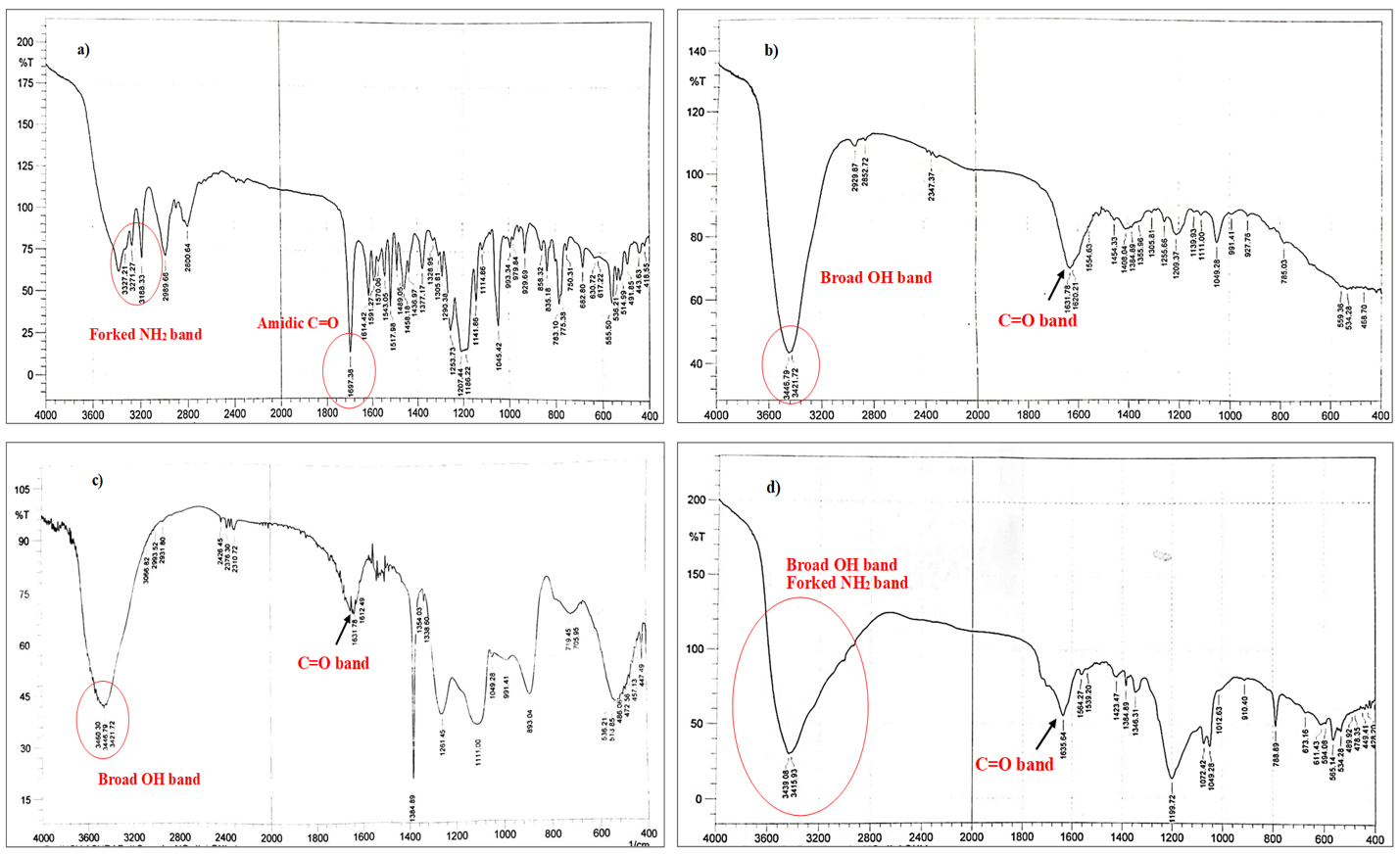


**Fig. S3.** The IR spectrum of SAF intact drug (a), SAF hydrolytic degradation product (b) and (c), and SAF oxidative degradation product (d).
